# Anxiety, depression, and stress: a comparative study between couples with male and female infertility

**DOI:** 10.1186/s12905-024-03072-5

**Published:** 2024-04-08

**Authors:** Zahra Bostani Khalesi, Fatemeh Jafarzadeh Kenarsari

**Affiliations:** Department of Midwifery, School of Nursing and Midwifery, Guilan University of Medical Sciences, Rasht, Iran

**Keywords:** Women, Men, Anxiety, Depression, Stress infertility

## Abstract

**Background and aim:**

Although infertility as a significant cause of marital crises is prevalent almost equally in men and women, infertile women are under more pressure and distress than infertile men. Therefore, this study was conducted aiming to compare anxiety, depression, and stress between couples with male and female infertility.

**Methods:**

In this descriptive-analytical cross-sectional study, 40 couples (*n* = 80) with male infertility and 40 couples (*n* = 80) with female infertility were referred to the infertility clinic of Al-Zahra Educational and Medical Center, Rasht, Iran. Eligible infertile couples were selected by convenience sampling method. The data collection tool was a two-part questionnaire consisting of a demographic information form and a short form of the standard Depression Anxiety Stress Scale-21 (DASS-21). Data analysis was carried out using descriptive and inferential statistical tests at a significant level of *p* < 0.05.

**Results:**

The severity of depression, anxiety, and stress also had a statistically significant difference between men and women. The severity of depression was mild in 57.5% of infertile women and moderate in 40% of infertile men. The severity of anxiety was moderate in 42.5% of infertile women and mild in 57.5% of infertile men. The severity of stress was Severe in 37.5% of infertile women and mild in 40% of infertile men. There was a statistically significant difference between infertile women and men in terms of depression (t=-4.213, df = 1619, *p* < 0.001), anxiety (t=-7.261, df = 2274, *p* < 0.001), and stress (t=-9.046, df = 2308, *p* < 0.001) subscales, and the total scores (t=-7.709, df = 2315, *p* < 0.001). The depression, stress and anxiety levels were higher in infertile women than in healthy women with infertile spouses. This difference was statistically significant (*p* < 0.01). The depression, anxiety, and stress levels were significantly different between infertile men and healthy men with infertile wives (*p* < 0.001).

**Conclusion:**

The results of this study indicated that depression, anxiety, and stress were more prevalent in infertile women than in infertile men. The severity levels of depression, anxiety, and stress in the wives of infertile men were higher than those in the spouses of infertile women.

## Introduction

Infertility is defined as at least 1 year of failed attempts by married partners at getting pregnant when the couple had been sexually active each month without contraception [1]. In developed countries, the prevalence of infertility is estimated to be 35% in both genders (43% in women and 30.7% in men), and the cause of infertility is unexplained or idiopathic in 5% of infertile couples [2]. The prevalence of primary infertility in Iran is 18.3%, of which 32% were reported to have inexplicable causes, 12.5% with female causes, and 13.6% with male causes [3].

Infertility has significant negative physical, psychological, and social impacts on the lives of infertile couples, particularly women [4]. The presence of other factors such as a T-shaped uterus [5], FIGO type 3 leiomyomas [6, 7] and process of fertility preservation for transgender men [8] may be a cause or concomitant cause of increased anxiety or depression in patients. A history of infertility is potential predictors of early and premature menopause in women [9]. Furthermore, Facing infertility treatment and potentially facing an altered future without children are stressful conditions that could adversely affect the mental health of couples [10]. Chronic stress suppresses immune system function and increases susceptibility to infection and many various diseases [11]. Anxiety and depression are reported to be more prevalent among women with infertility [12]. The overall prevalence of anxiety and depression in infertile couples has been estimated at 25-60%; this rate is significantly higher compared to fertile couples and the general population [13]. In Iran, more than 14% of women and 25% of men suffer from anxiety and stress. Although the symptoms of depression in women are 1.7 times more than that in men [14], research demonstrates that the prevalence of psychological disorders is 48-96% in infertile women, while 11.2% in fertile women [15].

Various studies worldwide have indicated that about 96% of infertile women undergo infertility stress in their lives [16]. The results of a study in Iran showed that 31.7% of infertile women had some levels of depression, and 15% had clinical depression; also, 20% of infertile men had some levels of depression, and 13.3% had clinical depression (15). Moreover, severe and very severe levels of anxiety were estimated at 36.7% and 61.6% in men and at 30% and 53.3% in women, respectively [14]. In other words, infertile men experience more anxiety than infertile women [10].

Anxious symptoms are considered among the most prevalent psychiatric problems in the general population of different societies, whose incidence rate is twice as high in women as in men [17]. Anxiety is a type of feeling of discomfort, fear, and worry, which can be a primary state or part of a response to stressors [18]. Besides the particular and cultural conditions existing among the people regarding the infertile individual, the high costs of artificial insemination methods and the mild odds of success are among other causes of high anxiety and depression of individuals with infertility [19].

Although infertility seems to be associated with couples, considering women’s sensitivity and vulnerability that is primarily due to their psychological and physical or social, cultural, and economic structures of societies, they are usually exposed to more negative effects [20]. Typically, women are considered more responsible for infertility, exposing them to psychological disorders [17]. Research demonstrates that even in infertility stemming from male factors, women usually face more family and social issues and problems, and it is women who tolerate the most burden of infertility [10].

Although numerous studies have been conducted aiming to determine the psychological consequences among infertile couples, most of these studies have dealt with comparing these negative consequences with fertile couples [11]. On the other hand, researchers comparing infertile women and men only in terms of psychological consequences have reported contradictory results; for example, the results of Pahlevani et al.’s [21] study indicated that infertile men experienced less stress than infertile women and enjoyed higher mental health. In comparison, according to Maroufizadeh et al.’s [16] study, anxiety was twice times higher in infertile women than in infertile men. Still, no statistically significant difference was found between these two groups in terms of the level of depression.

In Yousefi et al.’s 22(14) study, women who were the cause of infertility themselves had more anxiety than women with infertile spouses. In Riahi et al.’s [22] study, no significant gender difference was observed in the negative psycho-social consequences of infertility between infertile men and women.

Despite the discrepant results, during the research team’s extensive search, there were limited studies dealing with factors related to the psychological consequences of infertility. Therefore, this study was conducted aiming to compare anxiety, depression, and stress between couples with male and female infertility.

## Methods

### Participants, design, and procedure

In this descriptive-analytical cross-sectional study, 40 couples (*n* = 80) with male infertility and 40 couples (*n* = 80) with female infertility referring to the infertility clinic of Al-Zahra Educational and Medical Center, Rasht, Iran, between November 2022 and January 2023 were selected. The sample size was determined based on previous relevant studies. Sampling was performed using the convenience sampling method. The inclusion criteria included consent to participate, Iranian nationality, primary infertility confirmed by a gynecologist, lack of known psychological disorders (according to the participant’s statements), ability to read and write, having the physical ability to participate in research, not taking psychotropic drugs (self-report), no experience of stressful events (e.g., death of close relatives or job loss within the past 6 months), lack of narcotics abuse, and not having adopted children. The reason for unknown infertility, couples with both male and female infertility factors, and not completely responding to the questionnaire’s questions were exclusion criteria. Not completely responding to the questionnaire’s questions were exclusion criteria. After providing sufficient explanations about the study objectives, voluntary participation in the research, and the participant information’s confidentiality, all participants provided written informed consent.

### Measures

The data collection tool, the questionnaire, consisted of two parts. The first part was a researcher-made questionnaire that assessed demographic information to determine the participants’ individual-social characteristics (age, education level, occupation, ethnicity, age at marriage, economic status, place of residence, cause of infertility, family relationship with spouse, duration of knowing about infertility, duration of infertility treatment, and the number of infertility treatment times), which was completed by the researcher through an interview. The intended questionnaire was given to 10 faculty members to assess its formal validity, and after imposing the perspectives, the final modification was included in it.

The second part was the standard DASS-21 designed by Lavibound, containing two forms. The original form of this scale contains 42 phrases, in which each of the three subscales of depression, anxiety, and stress consists of 14 phrases. However, the short form of the original DASS-21 has 21 statements in three domains: Depression (including questions 3, 5, 10, 13, 16, 17, 21), anxiety (including questions 2, 4, 7, 9, 15, 19, 20), and stress (including questions 1, 6, 8, 11, 12, 14, 18), which in the current study, the short form of the original DASS-21 has been used. The depression subscale measures unhappy mood, lack of self-confidence, despair, worthlessness of life, lack of interest in involvement in affairs, lack of enjoyment of life, and lack of energy and power. The anxiety subscale evaluates physiological over-arousal, fears, and situational anxiety. Finally, the stress subscale encompasses difficulty achieving calmness, nervous tension, irritability, and restlessness. Participants were supposed to mark the severity of the symptom raised in each statement that they had experienced during the past week. The scoring method is in the form of a four-point Likert scale (never = 0, sometimes = 1, often = 2, and almost always = 3). Since each of the questionnaire short form’s subscales contains 7 questions, the final score of each subscale should be doubled. The final score of each subscale is obtained through the sum of the scores of the related questions.

Anthony et al. [23]. , approved its factor structure using exploratory factor analysis. The reliability of the scale for stress, anxiety, and depression was 0.81, 0.79, and 0.71, respectively. The reliability and validity of the short form of the test were confirmed by Sahebi et al. [24]. , in Iran.

The content validity ratio (CVR) and content validity index (CVI) of the DASS-21 were determined by eight faculty members. Internal consistency reliability and consistency reliability (test-retest) of the scale were determined using Cronbach’s alpha coefficient and the intraclass correlation coefficient (ICC), respectively. Internal consistency reliability and consistency reliability were calculated in 20 infertile individuals referring to Al-Zahra Educational and Medical Center at a two-week time interval. The total Cronbach’s alpha coefficient was equal to 0.890 for the DASS-21 and equal to 0.811, 0.738, and 0.847 for its depression, anxiety, and stress subscales, respectively, which was at the optimal level. The total ICC value was equal to 0.892 for the DASS-21 and equal to 0.856, 0.916, and 0.828 for its depression, anxiety, and stress subscales, respectively, which was at an optimal level. In order to obtain permission to use the mentioned questionnaires in this study, the main designers and researchers who conducted the psychometrics of the questionnaires in Iran were communicated electronically.

### Data analysis

Data for 40 couples with male factor infertility and 40 couples with female factor infertility were finally included in this study. Statistical Packages for Social Sciences (SPSS) Windows software, version 16 (IBM, Chicago IL, USA) was used in the analyses.

Descriptive statistics (Mean, standard deviation, ratio, and frequency values) were used to characterize the socio-demographics and clinical data of participants. Frequencies or percentages were used for categorical variables, and means with standard deviations or medians with interquartile ranges were used for continuous variables. Frequencies were compared between groups by chi-square test. Continuous variables were compared by the use of an independent t-test and are displayed as mean and standard deviation (SD).

The normality of the data of the continuous variables by using the Kolmogorov–Smirnov test was tested. Pearson’s chi-squared test was used analysis to compare categorical data between men and women. Independent-sample t-tests were used to examine the relationship between anxiety, depression, and stress symptoms with the socio-demographics of the two groups. The level of statistical significance for the comparison of the two groups was considered as *p* < 0.05.

## Results

In the current research, 40 infertile couples with a male factor and 40 infertile couples with a female factor completed the DASS-21. The mean age of female and male participants was 32.3 ± 1.74 and 37.6 ± 2.12, respectively; 50% of women had a university education level, and 55% of men had a diploma; 60% of women were housewives, and 60% of men were employees. The minimum and the maximum duration of infertility were 1 and 13 years, respectively and mean, 4.02 ± 1.49 years.

Comparing men and women demonstrated a significant statistical difference between the two groups in terms of age, occupation, and cigarette use (Table 1).

**Table 1:** Sociodemographic data of and clinical data the infertile men and women

Variable		Gender N (%)		P
		Male (40)	Female (40)	
**Age; Mean ± SD (year)**		37.6 ± 2.12	32.3 ± 1.74	0.001
**Duration of marriage; Mean ± SD (year)**		6.47 ± 5.31		
**Duration of infertility; Mean ± SD (year)**		4.02 ± 1.49		
**Duration of first medical advice; Mean ± SD (year)**		2.19 ± 1.07		
**Education level**	Elementary	7 (17.5)	2 (5)	NS
	High school	22 (55)	16 (40)	
	University	13 (32.5)	20 (50)	
**Occupation**	No qualified professions / Household	2 (5)	24 (60)	0.001
	Laborer	11(27.5)	4 (10)	
	Executive official	24 (60)	12 (30)	
	Management official	3 (7.5)	-	
**Location**	Rural	12 (30)		
	Urban	28 (70)		
**Smoking status**	Non smoker	11 (27.5)	33 (82.5)	0.001
	Occasional smoker	14 (35)	5 (12.5)	
	Regular smoker	15 (37.5)	2 (5)	
**Income status**	Income less than the expense	23 (57.5)		
	Expense equals income	12 (30)		
	Income more than the expense	5 (12.5)		
**Family history of psychiatric disorders**	Depressive Disorder	2 (5)	3(7.5)	NS
	Anxiety Disorder	1(2.5)	-	
	Other	1 (2.5)	-	
**History of ineffective treatments**	Yes	12 (30)		
	No	28 (70)		
**Ability to pay for the infertility treatment costs**	Yes	16 (40)		
	No	24 (60)		

^⁎^*P* < 0.05, NS: Non Significant

The mean scores of infertile women’s depression, anxiety, and stress were 5.53 ± 3.01, 4.36 ± 2.38, and 6.83 ± 3.02, respectively. Also, the mean scores of infertile men’s depression, anxiety, and stress were 4.62 ± 2.15, 3.28 ± 1.02, and 5.47 ± 2.41, respectively. There was a statistically significant difference between infertile men and women in terms of depression, anxiety, and stress subscales and the total scores (Table 2)

**Table 2:** Comparison of mean scores of depression, anxiety, and stress among infertile men and women

DASS-21	Gender (Mean ± SD)		t	df	p
	Male (40)	Female (40)			
Depression	4.62 ± 2.15	5.53 ± 3.01	t = -4.213	1619	< 0.001^*^
Anxiety	3.28 ± 1.02	4.36 ± 2.38	t = -7.261	2274	< 0.001^*^
Stress	5.47 ± 2.41	6.83 ± 3.02	t = -9.046	2308	< 0.001^*^
Total Score	13.40 ± 7.23	16.71 ± 10.55	t = -7.709	2315	< 0.001^*^

DASS-21: Depression-Anxiety-Stress Scale-21, t: Student-T test, ^⁎^: *p* ≤ 0.05

Furthermore, the severity of depression, anxiety, and stress also had a statistically significant difference between men and women. The severity of depression was mild in 57.5% of infertile women and moderate in 40% of infertile men. The severity of anxiety was moderate in 42.5% of infertile women and mild in 57.5% of infertile men. The severity of stress was Severe in 37.5% of infertile women and mild in 40% of infertile men (Table 3).

**Table 3:** Comparison of the Depression, anxiety, and stress among infertile men and women

DASS-21		Gender N (%)		χ^2^	*df*	*p*
		**Male (40)**	**Female (40)**			
**Depression**	Normal	11 (27.5)	7 (17.5)	31.258	4	< 0.001^*^
	Mild	17 (42.5)	23 (57.5)			
	Moderate	7 (17.5)	8 (20)			
	Severe	4 (10)	2 (5)			
	Extremely severe	1(2.5)	-			
**Anxiety**	Normal	9 (22.5)	3 (7.5)	29.216	4	< 0.001^*^
	Mild	23 (57.5)	11(27.5)			
	Moderate	6 (15)	17 (42.5)			
	Severe	2 (5)	6 (15)			
	Extremely severe	-	3(7.5)			
**Stress**	Normal	10 (25)	-	36.175	4	< 0.001^*^
	Mild	16 (40)	12 (30)			
	Moderate	3 (7.5)	7 (17.5)			
	Severe	1 (2.5)	15 (37.5)			
	Extremely severe	-	6 (15)			

DASS-21: Depression-Anxiety-Stress Scale-21, χ^*2*^: Chi-Square test, ^⁎^: *p* ≤ 0.05

The severity levels of depression, anxiety, and stress were also different between spouses of infertile women and wives of infertile men. More than 50% of spouses of infertile women had no symptoms of depression, while the severity of depression was mild in 40% of the wives of infertile men; 37.5% of the spouses of infertile women had no symptoms of anxiety, but the severity of anxiety was mild in 45% of wives of infertile men.

The severity of stress was mild in 55% of spouses of infertile women and moderate in 42.5% of wives of infertile men. The levels of stress and anxiety were higher in infertile women than in healthy women with infertile spouses. This difference was statistically significant, but the difference between the two groups in terms of depression was not significant. The levels of depression, anxiety, and stress had a statistically significant difference between infertile men and healthy men with infertile wives (Table 4).

**Table 4:** Comparison of the depression, anxiety, and stress among the wives of infertile men and the spouses of infertile women

DASS-21		The wives of infertile men (40)	The spouses of infertile women(40)	χ^2^	df	p
**Depression**	Normal	10 (25)	21 (52.5)	30.114	4	< 0.001^*^
	Mild	16 (40)	11 (27.5)			
	Moderate	12 (30)	7 (17.5)			
	Severe	2 (5)	1 (2.5)			
	Extremely severe	-	-			
**Anxiety Severity**	Normal	5 (12.5)	15 (37.5)	32.137	4	< 0.001^*^
	Mild	18 (45)	13 (32.5)			
	Moderate	14 (35)	11 (27.5)			
	Severe	2 (5)	1 (2.5)			
	Extremely severe	1 (2.5)	-			
**Stress Severity**	Normal	1 (2.5)	8 (20)	35.228	4	< 0.001^*^
	Mild	14 (35)	22 (55)			
	Moderate	17 (42.5)	6 (15)			
	Severe	5 (12.5)	3 (7.5)			
	Extremely severe	3 (7.5)	1 (2.5)			

DASS-21: Depression-Anxiety-Stress Scale-21, χ^*2*^: Chi-Square test, ^⁎^: *p* ≤ 0.05

## Discussion

The results of the current research indicated that infertility accompanied depression, anxiety, and stress in both women and men and their spouses, the mean scores of depression, anxiety, and stress were more severe in infertile women than in infertile men. Numerous studies have reported a severe prevalence of anxiety, and depression, and treatments such as the beneficial effects of the clinical use of myo-ins among infertile couples [11, 16, and 25]. In their study, Haimovici et al. [26]. , reported that both women and men experienced depression and anxiety stemming from infertility, but women showed severe severity of clinical symptoms. Chiaffarino et al. [27]. , found that infertile women experienced more anxious and depressive symptoms than infertile men. Moreover, Amini et al. [15]. , reported in their study that mental health problems were considerably more prevalent in infertile women than in infertile men. Alosaimi et al. [12]. , also found that infertile couples had significantly severer scores on depression than the control group.

Fertility in traditional societies, such as Iran, is considered a part of women’s responsibility, which can justify severe levels of depression, anxiety, and stress in infertile women than in infertile men [28]. According to a study in Japan, women often consider themselves responsible for infertility. Also, Irani et al. [29]. , reported that infertile women experienced more feelings of inferiority and incompetence than infertile men.

In numerous studies, researchers have attempted to explain the psychopathological mechanisms of anxiety and depression in infertile couples. They assumed that coping adaptive strategies were different based on gender so that in most cases, women considered themselves responsible for infertility and further regarded infertility as a threat and harm compared to men, leading to increasing their distress. These gender differences in psychopathology mechanisms could explain the consistency between our and the other results found in other countries [30]. Socio-cultural factors are just one of the numerous factors contributing to coping strategies, but they cannot explain all the factors [31].

Our study results revealed that the severity levels of depression, anxiety, and stress were higher in the wives of infertile men than in the spouses of infertile women. Moreover, infertile women had more severe levels of anxiety and stress than healthy women with infertile spouses.

Our study results revealed that the severity levels of depression, anxiety, and stress were higher in the wives of infertile men than in the spouses of infertile women. Moreover, infertile women had more severe levels of anxiety and stress than healthy women with infertile spouses. Amini et al. [15]. , also reported a higher prevalence of anxiety and depression in women, regardless of the cause of infertility (male or female factor).

The present research demonstrated that the level of depression was more severe in half of the infertile women and about one-third of infertile men than in healthy women and men, one of the reasons for which could be the problems for getting pregnant and the family pressure on the couple to get pregnant; this infertility psychological burden can influence the infertile couple’s whole life [10]. The fear of an uncertain future and failed infertility treatment, severe treatment costs, and its consequences are annoying for many women and men, and most of these problems have been neglected by the treatment team and the health field [32]. A deep look at infertile women and men’s statements denotes that they need to be supported by their spouses, those around them, the medical team, and insurance services [33].

### Limitations

The study could be a reference for developing psychological and counseling intervention protocols for these couples. One other of the strengths of the present research was the equal number of infertile participants with male and female factors. Also, the infertility factor was taken into account as an important factor considered when investigating the psychological effect of infertility based on gender. The current study had limitations, such as the small sample size and not determining the association of the severity of depression, anxiety, and stress with the duration of infertility and the number of failed infertility treatments. The study was limited to infertile males and females who were seeking infertility treatment. Therefore, infertile couples who had not tried to receive treatment were not investigated.

## Conclusion and implications

The results of this study demonstrated that infertility accompanied depression, anxiety, and stress in both women and men. Although this mental distress was severer in infertile women than in infertile men, the severity levels of depression, anxiety, and stress were severer in the wives of infertile men than in the spouses of infertile women. Also, infertile women had severer levels of anxiety and stress than healthy women with infertile spouses. The levels of depression, anxiety, and stress were severer in infertile men than in healthy men with infertile wives. Therefore, infertility is not only considered a physical problem but also affects the psycho-social health of infertile individuals and their spouses.

Although our data support the conclusion that infertility can have a greater impact on women’s mental health than on men, more longitudinal studies with larger sample sizes are required to specify mental health changes based on gender and to control for other factors in order to plan intervention strategies by considering the psychological support needed by infertile couples.

## Data Availability

The datasets used and analyzed during the current study are available for ge.
