# Correction: Anxiety, depression, and stress: a comparative study between couples with male and female infertility

**DOI:** 10.1186/s12905-025-03589-3

**Published:** 2025-02-11

**Authors:** Zahra Bostani Khalesi, Fatemeh Jafarzadeh Kenarsari

**Affiliations:** Department of Midwifery, School of Nursing and Midwifery, Guilan University of Medical Sciences, Rasht, Iran

**Correction:** **BMC Women’s Health 24, 228 (2024)**

**https://doi.org/10.1186/s12905-024-03072-5**

In the original publication of this article [1], the sentence beginning ‘In this descriptive-analytical cross-section... were selected.' in *Participants, design, and procedure* under *Method* section of this article, the month and year 'November 2021 and January 2022' should have read correctly as 'November 2022 and January 2023'. The incorrect and corrected sentence is given below. The original article has been corrected.

Incorrect sentence

In this descriptive-analytical cross-sectional study, 40 couples (n = 80) with male infertility and 40 couples (n = 80) with female infertility referring to the infertil¬ity clinic of Al-Zahra Educational and Medical Center, Rasht, Iran, between November 2021 and January 2022 were selected.

Correct sentence

In this descriptive-analytical cross-sectional study, 40 couples (n = 80) with male infertility and 40 couples (n = 80) with female infertility referring to the infertil¬ity clinic of Al-Zahra Educational and Medical Center, Rasht, Iran, between November 2022 and January 2023 were selected.

## References

[1] Khalesi ZB, Kenarsari FJ. Anxiety, depression, and stress: a comparative study between couples with male and female infertility. BMC Women’s Health. 2024;24:228. 10.1186/s12905-024-03072-5. 38589804 10.1186/s12905-024-03072-5 PMC11003146

